# Coagulation Factor XII Levels and Intrinsic Thrombin Generation in Multiple Sclerosis

**DOI:** 10.3389/fneur.2018.00245

**Published:** 2018-04-20

**Authors:** Nicole Ziliotto, Marcello Baroni, Sofia Straudi, Fabio Manfredini, Rosella Mari, Erica Menegatti, Rebecca Voltan, Paola Secchiero, Paolo Zamboni, Nino Basaglia, Giovanna Marchetti, Francesco Bernardi

**Affiliations:** ^1^Department of Life Sciences and Biotechnology, University of Ferrara, Ferrara, Italy; ^2^Neuroscience and Rehabilitation Department, Ferrara University Hospital, Ferrara, Italy; ^3^Department of Biomedical and Specialty Surgical Sciences, University of Ferrara, Ferrara, Italy; ^4^Hematology Section, Department of Medical Sciences, Centre for Hemostasis and Thrombosis, University of Ferrara, Ferrara, Italy; ^5^Department of Morphology, Surgery and Experimental Medicine, University of Ferrara, Ferrara, Italy; ^6^LTTA Centre, University of Ferrara, Ferrara, Italy

**Keywords:** multiple sclerosis, coagulation, factor XII, intrinsic pathway, thrombin generation

## Abstract

**Background:**

Factor XII (FXII) activation initiates the intrinsic (contact) coagulation pathway. It has been recently suggested that FXII could act as an autoimmunity mediator in multiple sclerosis (MS). FXII depositions nearby dentritic cells were detected in the central nervous system of MS patients and increased FXII activity has been reported in plasma of relapsing remitting and secondary progressive MS patients. FXII inhibition has been proposed to treat MS.

**Objective:**

To investigate in MS patients multiple FXII-related variables, including the circulating amount of protein, its pro-coagulant function, and their variation over time. To explore kinetic activation features of FXII in thrombin generation (TG).

**Methods:**

In plasma from 74 MS patients and 49 healthy subjects (HS), FXII procoagulant activity (FXII:c) and FXII protein (FXII:Ag) levels were assessed. Their ratio (FXII:ratio) values were derived. Intrinsic TG was evaluated by different triggers.

**Results:**

Higher FXII:Ag levels (*p* = 0.003) and lower FXII:ratio (*p* < 0.001) were detected in MS patients compared with HS. FXII variables were highly correlated over four time points, which supports investigation of FXII contribution to disease phenotype and progression. A significant difference over time was detected for FXII:c (*p* = 0.031). In patients selected for the lowest FXII:ratio, TG triggered by ellagic acid showed a trend in lower endogenous thrombin potential (ETP) in MS patients compared with HS (*p* = 0.042). Intrinsic triggering of TG by nucleic acid addition produced longer time parameters in patients than in HS and substantially increased ETP in MS patients (*p* = 0.004) and TG peak height in HS (*p* = 0.008). Coherently, lower FXII:ratio and longer lag time (*p* = 0.02) and time to peak (*p* = 0.007) point out a reduced response of FXII to activation in part of MS patients.

**Conclusion:**

In MS patients, factor-specific and modified global assays suggest the presence of increased FXII protein level and reduced function within the intrinsic coagulation pathway. These novel findings support further investigation by multiple approaches of FXII contribution to disease phenotype and progression.

## Introduction

Multiple sclerosis (MS) is a chronic autoimmune disorder, characterized by immune-mediated inflammation and multifocal demyelinated lesions within the central nervous system (CNS) (1, 2). Growing evidences suggest the crosstalk between hemostasis components, inflammation, and immune system, which appear to be involved in MS pathophysiology (3–8). The relationship of the coagulation pathway with disease processes could be further supported by recent findings, showing that anticoagulation ameliorated clinical course of experimental autoimmune encephalomyelitis (EAE), an animal model of MS (9, 10).

Among coagulation factors, a key role seems accomplished by factor XII (FXII), the initiator of the “old” contact (intrinsic) coagulation pathway (11), which cooperates redundantly with the extrinsic pathway, thus re-defining the cascade model [reviewed in Ref. (12)]. Upon contact with negative charged surfaces, including nucleic acids (NAs) released by damaged cells, zymogen FXII is converted to activated FXII (FXIIa), which starts the sequential proteolytic reactions within coagulation cascade with activation of factor XI, and subsequent thrombin generation (TG) and final fibrin formation. Moreover, FXII regulates fibrinolysis, complement activation, and the kallikrein–kinin pathway (11, 13).

Recently, histological analysis of CNS tissue from MS patients identified FXII light chain depositions nearby dendritic cells (DCs) (14). The basic FXII protein structure consists of an N-terminal heavy chain with six domains for substrates interaction, and a C-terminal light chain, which includes the catalytic domain (15).

In EAE, FXII depletion had protective effects, reducing susceptibility to CNS inflammation, delaying disease onset, decreasing disease severity and production of T helper 17 (Th17) cells (14). In mice, FXII stimulates expression of CD87 receptor on DCs, which is crucial for inducing Th17 cells differentiation. To note, both FXIIa and zymogen FXII forms were found to modulate conventional DCs function inducing excessive production of cytokines during neuroinflammation in CNS. In support of these findings, deficiency of factor XI, directly activated by FXIIa, did not alter the disease course in EAE model.

These results, indicating that FXII could not contribute to the MS animal model through the intrinsic coagulation pathway, suggest that the FXII procoagulant activity “*per se*” is not involved in MS. Nevertheless, the FXII levels in plasma are usually assessed by a procoagulant assay (FXII:c). In fact, FXII:c has been found significantly increased in patients with relapsing-remitting MS (RR-MS) and secondary progressive MS (SP-MS) compared with healthy donors. Additionally, enhanced FXII:c was associated with relapses and shorter relapse-free period, independently from immune-modulatory therapy (14).

It has been proposed that FXII inhibition could represent a new approach in MS therapy, as indicated by the reduced number and severity of relapses in the EAE mouse model by injection of a recognized FXII inhibitor (14, 16). This would probably not cause bleeding tendency in patients, because it is well known that FXII deficiency does not compromise effective hemostasis (17, 18).

Interestingly, clinical and genetic reports point at a FXII role in thrombosis (19–21). Local and systemic thrombotic events has been described in MS potentially in relation to the overstimulation of innate immunity for both its inflammatory and coagulant components (22). Recently, the hypercoagulability and potentially prothrombotic state in MS patients has been investigated by TG, triggered by extrinsic activation (23).

The poorly defined role of FXII forms and features and paucity of studies in patients, strongly support investigation of FXII in MS. We investigated multiple FXII-related variables, as well as FXII activation in the intrinsic TG, to explore their association with MS.

## Materials and Methods

### Study Population

The study population included MS patients, the majority of which participated in the RAGTIME study (https://ClinicalTrials.gov ID:NCT02421731) (24). This clinical trial compares robot-assisted gait training vs. conventional therapy on mobility in severely disabled progressive MS patients.

All MS patients underwent to neurological visits, MRI examinations, and assessment of the Expanded Disability Status Scale (EDSS).

Inclusion and exclusion criteria for RAGTIME study were previously reported (24). The selection criteria for the present study included: age between 18 and 79 years, MS diagnosis according to the revised McDonald criteria (25), lack of MS worsening in the previous 3 months.

The healthy subjects (HS) group was represented by healthy volunteers, who were never diagnosed with MS, neurological disorder, other chronic inflammatory disease, and cardiovascular disease.

All subjects were of Caucasian origin. Patients were not under treatment with anticoagulant drugs. Written informed consent was obtained from all subjects, and the study was approved by the Ethical Committee of the S. Anna University-Hospital, Ferrara, Italy. The demographic and clinical characteristics of the study populations, which included 74 MS patients (12 relapsing remitting, RR-MS; 28 primary progressive PP-MS; 34 secondary progressive SP-MS) and 49 HS, are summarized in Table 1. Age was significantly different between MS and HS (*p* < 0.001, Student’s *t*-test, Table 1), while gender difference was not significant (*p* = 0.138, Fisher’s exact test). The total number of patients under disease-modifying treatments (DMTs) at blood sampling was 12 (5 patients under DMTs and 7 patients under both DMTs and symptomatic treatments), as detailed in Table 1. Five patients (three RR-MS and two SP-MS) with discontinuation of DMTs before their enrollment in the present study were included in the group “None treatment,” since at sampling they were not under treatment.

**Table 1:** Demographic and clinical characteristics.

	All MS	RR-MS	SP-MS	PP-MS	HS
Sample size, *n*	74	12	34	28	49
Female, *n* (%)	48 (64.9)	8 (66.7)	19 (55.9)	21 (75)	25 (51)
Age, mean ± SD	53.5 ± 10.7	43.5 ± 9	52.2 ± 8.9	59.3 ± 9.9	40.6 ± 13.3
EDSS, median (IQR)	6 (0.5)	3 (2)	6.5 (0.5)	6 (0.5)	–
Disease duration, mean ± SD	14.4 ± 10.0	7.4 ± 5.2	18.0 ± 8.1	13.5 ± 11.7	–
Treatment, *n* (%)					
Disease-modifying	5 (6.8)	–	1 (2.9)	4 (14.3)	–
Symptomatic	17 (22.9)	–	7 (20.6)	10 (35.7)	
Both	7 (9.5)	1 (8.3)	5 (14.7)	1 (3.6)	
None	45 (60.8)	11 (91.7)	21 (61.8)	13 (46.4)	

*MS, multiple sclerosis; RR-MS, relapsing-remitting multiple sclerosis; SP-MS, secondary progressive multiple sclerosis; PP-MS, primary progressive multiple sclerosis; HS, healthy subjects; n, number; EDSS, Expanded Disability Status Scale; IQR, interquartile range*.

*Age and disease duration in years are reported as mean ± SD. For the ordinal EDSS, the median (interquartile range) is given*.

*Disease-modifying treatments were as follow: 1 (RR) Rituximab; 1 (SP) interferon-beta; 1 (SP) glatiramer acetate; 1 (SP) methotrexate; 1 (SP) teriflunomide; 2 (1 SP and 1 PP) fingolimod; 2 (1 SP and 1 PP) azathioprine; 2 (PP) natalizumab; 1 (PP) cyclophosphamide*.

*Symptomatic treatments were as follow: 16 (1 RR, 6 SP, and 9 PP) oral baclofen; 2 (SP) Pregabalin; 1 (SP) oral baclofen plus gabapentin; 1 (SP) tetrahydrocannabinol plus cannabidiol; 1 (SP) combination of clonazepam, tetrahydrocannabinol and cannabidiol; 2 (1 SP and 1 PP) oral baclofen plus amitriptyline; 1 (PP) amantadine*.

*Descriptive analysis between MS and HI were performed using Fisher’s exact test and Student’s t-test*.

### Plasma Samples

Venous peripheral blood samples from both MS patients and HS were collected into sodium citrate tubes. Patients enrolled in the RAGTIME study provided blood sampling at four time point: (T0) baseline point, prior to the first rehabilitative session; (T1) intermediate point, after six training sessions; (T2) end of treatment, 12 completed rehabilitative sessions, 1 month after T0; (T3) follow-up, after 3 months from the end of training program. Plasma samples were obtained after two consecutive centrifugations of blood samples, at room temperature (2,500 *g* for 15 min and 11,000 *g* for 5 min). Aliquots were stored at −80°C until use.

### FXII Activity

Coagulant activity of FXII (FXII:c) in plasma samples was assessed by an activated partial thromboplastin time (aPTT)-based assay (HemosIL aPTT SynthASil kit, Instrumentation Laboratory, Lexington, MA, USA). Activity and coagulation times were recorded by the ACLTOP 700 instrument (HemosIL, Instrumentation Laboratory). The inter-assay coefficients of variation assessed over multiple runs was 2.1%.

### FXII Antigen

Plasma FXII antigen (FXII:Ag) concentrations were determined using a sandwich enzyme-linked immunosorbent assay kit (LS-F10418, LifeSpan Biosciences, Seattle, WA, USA), following the manufacturer’s instructions. The assay uses a polyclonal capture antibody for FXII and a mouse primary monoclonal antibody raised against the heavy chain of FXII as detection antibody. The plasma samples were tested with a dilution of 1:3,000. The results were expressed as relative units in percentage generated from concentration values normalized to a pool of normal plasma loaded in all plates. The inter-assay coefficient of variation for plasma measurements was 2.6%.

### Intrinsic TG

Thrombin generation in plasma samples was evaluated by the addition of a specific thrombin fluorogenic substrate (Calbiochem-Novobiochem, La Jolla, CA, USA) (26, 27). Plasma samples were diluted (1/5) in a HBS buffer (Hepes 20 mM, NaCl 150 mM, PEG-8000 0.1%, pH 7.4) and incubated for 5 min at 37°C. TG through intrinsic activation was conducted by addition of a volume mixture of ellagic acid (Dade Actin FS, Siemens) and phospholipid vesicles (4 µM, MP-reagent, Stago), as previously reported (28, 29). TG was also evaluated by further addition of NA as trigger (1 µM) for the activation. Final concentrations of CaCl_2_ and thrombin fluorogenic substrate were 2.5 mM and 250 µM, respectively. The fluorescence was measured overtime in a fluorometer (Fluoroskan Ascent BioMed) and the amount of the generated thrombin was calculated using a normal pooled human plasma (Hyphen BioMed) as a standard. As negative control of contact activation, the FXII inhibitor “corn trypsin inhibitor” was added to the normal pooled plasma in each assay condition (single/double trigger).

Specific parameters of TG-lag time, time to peak (TTP), peak height, and endogenous thrombin potential (ETP) (area under the curve) were obtained by a nonlinear regression analysis of the first derivative of relative fluorescence units using the software version 6.01 (GraphPad Software, Inc., La Jolla, CA, USA).

### Statistical Analysis

All statistical analyses were performed using IBM^®^ SPSS^®^ Statistics version 24 software (IBM Corp., Armonk, NY, USA) and figures were produced by GraphPad Prism version 6.01 (GraphPad Software, Inc., La Jolla, CA, USA).

The Shapiro–Wilk test was used to test for normality of continuous variables. The Fisher’s exact test was used to compare differences in categorical variables and Student’s *t*-test was used to compare age between total MS and HI groups.

Comparisons of MS vs. HS and of males vs. females of FXII:c, FXII:Ag, and FXII:c/FXII:Ag ratio (FXII:ratio) were conducted with the ANCOVA test using age as covariate. Comparisons for FXII levels among clinical subgroups were performed with ANCOVA test using age as covariate and, in case of a significant *p*-value, pairwise comparisons were Bonferroni corrected for multiple testing (*q*-values).

To assess whether FXII levels were significantly different among patients receiving DMTs, symptomatic treatments, or none current treatment, one-way ANOVA was used and, in case of a significant *p*-value, pairwise comparisons were Bonferroni corrected for multiple testing (*q*-values).

Pearson’s test was used to assess correlation over time for FXII:c and FXII:Ag. ANOVA for repeated measures was used to test FXII:c, FXII:Ag, and FXII:ratio across the four time points and, in case of a significant *p*-value, pairwise comparisons were Bonferroni corrected (*q*-values). Student’s *t*-test was used to compare TG parameters of MS patients with those of HS, while paired Student’s *t*-test was used to assess differences in TG after NA addition in MS and HS.

## Results

### FXII Activity and Antigen Levels

Factor XII coagulant activity (FXII:c), FXII protein concentration (FXII:Ag), and their ratio, providing quantitative information about the FXII activity in relation to the amount of circulating protein, were evaluated in plasma of MS patients and of HS and summarized in Table 2. Comparison between MS and HS groups revealed significant differences in FXII:Ag (*p* = 0.003) and FXII:ratio (*p* < 0.001) but not in FXII:c (*p* = 0.421). No differences within clinical subgroups (RR-MS, SP-MS, and PP-MS) were detected for FXII:c (*p* = 0.296), FXII:Ag (*p* = 0.248), and FXII:ratio (*p* = 0.765). Comparison between male and female within MS and HS groups, after age adjustment, did not reveal difference in FXII:c (MS *p* = 0.74, HS *p* = 0.374), FXII:Ag (MS *p* = 0.256, HS *p* = 0.622), and showed a trend in difference in FXII:ratio in HS (*p* = 0.045), but not in MS (*p* = 0.11).

**Table 2:** FXII activity, antigen, and ratio in MS patients and HS.

	MS						HS			MS vs. HS
			
		Female	Male	RR	PP	SP		Female	Male	*p*-value
			
*N*	74	48	26	12	28	34	49	25	24	
**FXII:c %**										0.421
Mean	115.0	113.9	116.9	111.3	115.0	116.2	123.7	127.5	119.8	
Lower 95% CI	110.3	108.7	107.1	97.3	108.5	108.3	116.5	115.3	111.7	
Upper 95% CI	119.7	119.1	126.7	125.2	121.5	124.1	130.9	139.8	127.8	
**FXII:Ag %**										0.003
Mean	106.7	102.7	114.2	106.2	108.1	105.8	99.3	99.0	99.6	
Lower 95% CI	99.3	93.6	101.0	85.3	95.2	94.8	91.4	85.5	90.5	
Upper 95% CI	114.1	111.7	127.4	127.1	121.0	116.8	107.2	112.5	108.8	
**FXII:ratio**										<0.001
Mean	1.14	1.18	1.06	1.09	1.15	1.15	1.30	1.36	1.24	
Lower 95% CI	1.07	1.09	0.98	0.96	1.02	1.06	1.22	1.23	1.14	
Upper 95% CI	1.21	1.28	1.13	1.21	1.27	1.25	1.38	1.48	1.33	

*MS, multiple sclerosis; HS, healthy subjects; RR-MS, relapsing-remitting multiple sclerosis; SP-MS, secondary progressive multiple sclerosis; PP-MS, primary progressive multiple sclerosis; FXII:c, factor XII activity; FXII:Ag, factor XII antigen; FXII:ratio, FXII:c/FXII:Ag; CI, confidence interval; N, number*.

*Analysis were conducted with the ANCOVA test, using age as covariate*.

The potential modulation of FXII levels by treatments was investigated. Patients under both DMTs and symptomatic treatments (Table 1) were categorized in DMTs group for the purpose of the analyses. No difference according to DMTs, symptomatic treatments, or none current treatments were detected for FXII:c (*p* = 0.98), FXII:Ag (*p* = 0.81), and FXII:ratio (*p* = 0.97).

A large portion of patients under study were characterized by a small range of EDSS (6–6.5), which does not favor the investigation of the relation between EDSS and FXII levels.

Factor XII:c, FXII:Ag, and FXII:ratio levels were also investigated over four time points (Table 3) in 49 MS (23 PP-MS and 26 SP-MS). A significant difference over time was detected for FXII:c (*p* = 0.031, Table 3). In particular, pairwise analysis revealed differences between T0 and T1 (*p* = 0.004; *q* = 0.023) and T0–T3 (*p* = 0.005; *q* = 0.027). The potential influence on FXII:c over time variations of MS phenotype or drug treatments was investigated. Differences were not detected within each clinical MS group (SP-MS, *p* = 0.079; PP-MS, *p* = 0.093), as well as within drug treatment groups (DMTs, *p* = 0.188; symptomatic treatment, *p* = 0.345; none, *p* = 0.142).

**Table 3:** FXII activity, antigen, and ratio in multiple sclerosis patients over four time points.

	Time points				
		
*N 49*	T0	T1	T2	T3	*p*-value
**FXII:c %**					0.031
Mean	114.5	110.4	114.1	110.0	
Lower 95% CI	108.6	104.5	107.3	103.8	
Upper 95% CI	120.4	116.3	120.8	116.2	
**FXII:Ag %**					0.596
Mean	105.1	107.8	108.8	105.8	
Lower 95% CI	95.87	98.06	99.54	96.87	
Upper 95% CI	114.4	117.5	118.0	114.8	
**FXII:ratio**					0.151
Mean	1.16	1.10	1.10	1.09	
Lower 95% CI	1.07	1.01	1.03	1.02	
Upper 95% CI	1.25	1.18	1.17	1.16	

*FXII:c, factor XII activity; FXII:Ag, factor XII antigen; FXII:ratio, FXII:c/FXII:Ag; CI, confidence interval; N, number*.

*ANOVA for repeated measures was used to test FXII:c, FXII:Ag, and FXII:ratio across the four time points*.

No differences over time were detected for FXII:Ag (*p* = 0.596) and FXII:ratio (*p* = 0.151) (Table 3).

Noteworthy, analysis of correlations among time points for each FXII parameter (Table 4) showed that FXII:c levels were highly correlated (T0–T1, *r*^2^ = 0.90; T1–T2, *r*^2^ = 0.82; T2–T3, *r*^2^ = 0.75; *p* < 0.001) as well as FXII:Ag levels (T0–T1, *r*^2^ = 0.81; T1–T2, *r*^2^ = 0.79; T2–T3, *r*^2^ = 0.84; *p* < 0.001).

**Table 4:** Correlations of factor XII activity and antigen over four time points in multiple sclerosis patients.

	T0	T1	T2
FXII:c			
**T1**	0.90		
**T2**	0.80	0.82	
**T3**	0.88	0.84	0.75
FXII:Ag			
**T1**	0.81		
**T2**	0.81	0.79	
**T3**	0.63	0.70	0.84

*Pearson’s test was used to assess correlation over time for factor FXII activity (FXII:c) and antigen (FXII:Ag). (T0) baseline point, prior to the first rehabilitative session; (T1) intermediate point, after six training sessions; (T2) end of training, 12 completed rehabilitative sessions, 1 month after T0; (T3) follow-up, after 3 months from the end of training program*.

### Intrinsic TG

**Figure 1:**
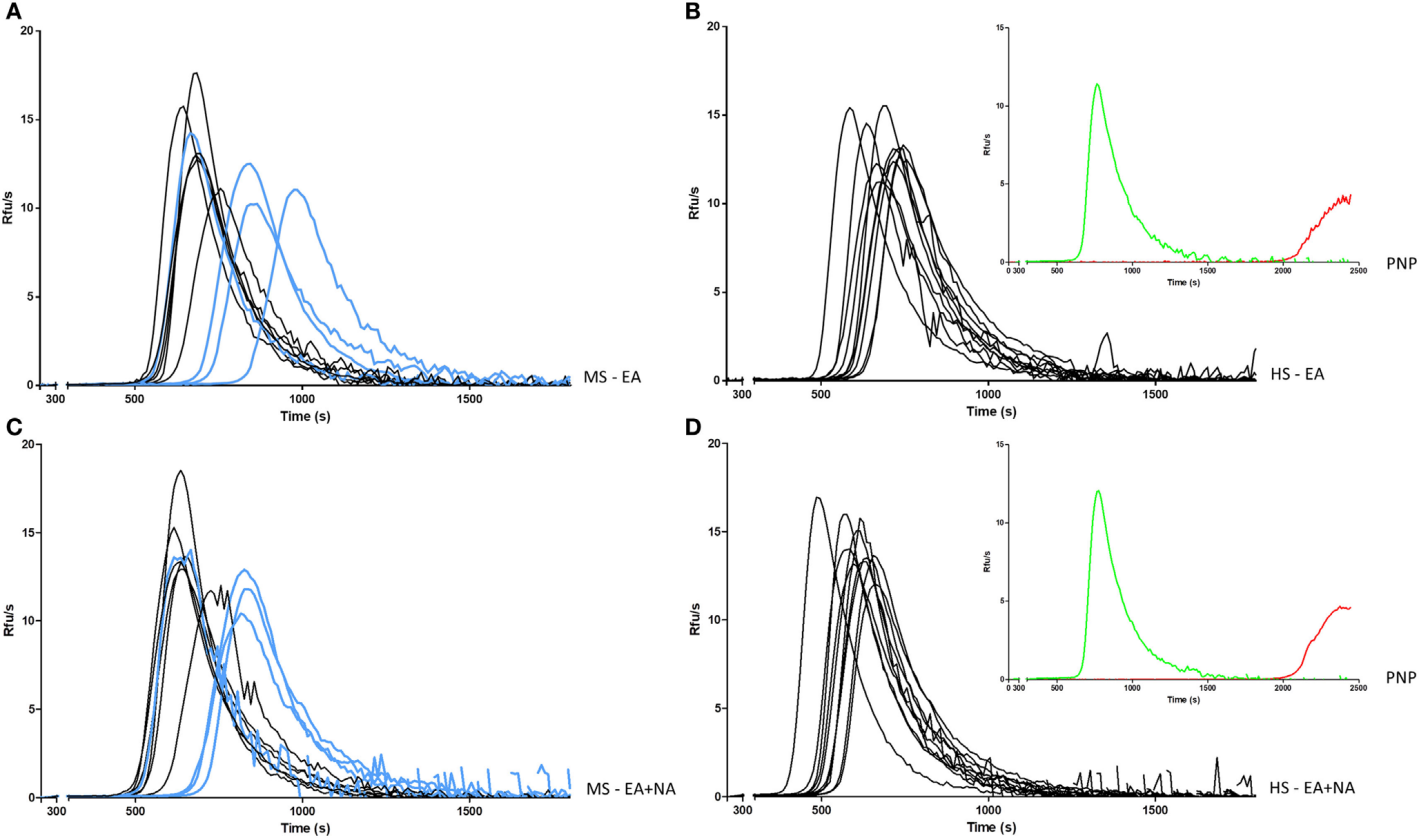
Intrinsic generation of thrombin in plasma of MS patients and HS. Thrombin generation activity in plasma samples was triggered by EA in MS patients **(A)** and in HS **(B)**, or EA plus NA in MS patients **(C)** and in HS **(D)**. Curves from PP-MS are shown in light blue. Curves of PNP (insets) in the same conditions of MS patients and HS (green), and after addition of FXII inhibitor (red) are reported as control. MS, multiple sclerosis; PP-MS, primary progressive multiple sclerosis; HS, healthy subjects; EA, ellagic acid; NA, nucleic acid; PNP, pooled normal plasma; RFUs, relative fluorescence units; s, seconds.

The decreased FXII:ratio values in MS patients prompted us to investigate potential variation of FXII specific activity through a global plasma assay (TG), which describes all phases of coagulation process and the integrated amount of generated thrombin (30). In particular, to provide kinetic information about the coagulation pathway triggered by intrinsic activation, a single classic activation (ellagic acid) was conducted in parallel with a double activation (Figure 1), by adding as trigger molecules of NA. This natural substance, released after cell death, is able to activate the contact pathway (31, 32).

To magnify FXII-related differences in TG, 10 patients’ plasma, obtained at T0 (4 PP-MS, 6 SP-MS), were selected for the lowest FXII:ratio (≤0.93), virtually undetectable in HS, and compared with 10 HS plasma with the highest FXII:ratio (≥1.4), which on the other hand was rare in MS patients.

Thrombin generation curves and parameters are reported in Figure 1 and in Table 5, respectively. TG activated by ellagic acid showed only a trend in lower thrombin potential (ETP) in MS patients compared with HS (2,631 ± 166 vs. 2,780 ± 136, *p* = 0.042).

**Table 5:** Generation of thrombin in plasma of multiple sclerosis patients and HS.

							EA vs. EA + NA	
	EA			EA + NA			*p*-Value	
			
	MS	HS	*p*-Value	MS	HS	*p*-Value	MS	HS
Lag time (s)	612 ± 97	564 ± 44	0.175	561 ± 81	487 ± 44	0.02	0.006	<0.0001
TTP (s)	750 ± 109	690 ± 51	0.132	706 ± 91	605 ± 51	0.007	0.014	<0.0001
Peak (RFU/s)	13.1 ± 2.3	13.4 ± 1.4	0.804	13.5 ± 2.2	14.3 ± 1.5	0.335	0.153	0.008
ETP (RFU)	2,631 ± 166	2,780 ± 136	0.042	2,748 ± 133	2,746 ± 38	0.964	0.004	0.506

*MS, multiple sclerosis; HS, healthy subjects; ETP, endogenous thrombin potential; EA, ellagic acid; NA, nucleic acid; TTP, time to peak; RFUs, relative fluorescence units; s, seconds*.

*Student’s *t*-test was used to compare TG parameters of MS patients with those of HS, while paired Student’s *t*-test was used to assess differences in TG after NA addition in MS and HS (*p*-value EA vs. EA + NA)*.

The TG triggered, in the same experiment, with the addition of NA, produced a clear decrease in main time parameters both in MS patients and in HS. As compared with the single ellagic acid trigger, both lag time and TTP were shorter in MS patients (612 ± 97 vs. 561 ± 81, *p* = 0.006; 750 ± 109 vs. 706 ± 91, *p* = 0.014, respectively) and in HS (564 ± 44 vs. 487 ± 44, *p* < 0.0001; 690 ± 51 vs. 605 ± 51, *p* < 0.0001). After the double induction, the increase in thrombin peak height and ETP differed between patients and HS. Particularly, the ETP value increased only in MS patients (2,631 ± 166 vs. 2,748 ± 133, *p* = 0.004), whereas the peak height was significantly increased only in HS (13.4 ± 1.4 vs. 14.3 ± 1.5, *p* = 0.008).

**Figure 2:**
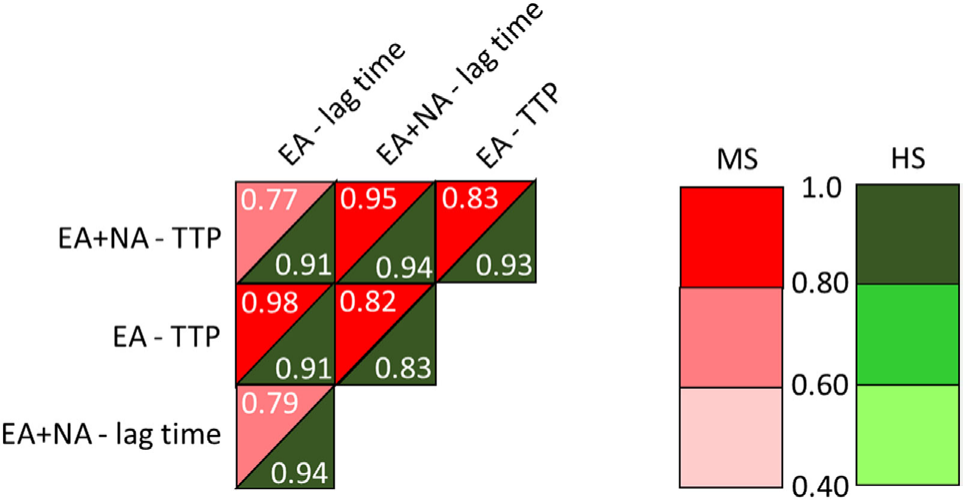
Correlations between time parameters from the intrinsic thrombin generation (TG) triggered by EA or by ellagic plus NA. MS, multiple sclerosis; HS, healthy subjects; EA, ellagic acid; NA, nucleic acid; TTP, time to peak. Linear models were generated to determine the relationship between TG parameters.

The comparison between MS patients and HS of TG, after the double trigger, showed longer time parameters in MS patients. Lag time was longer as a trend (561 ± 81 vs. 487 ± 44 in HS, *p* = 0.02) and TTP was around 100 s longer (706 ± 91 vs. 605 ± 51 in HS, *p* = 0.007). To note, three out of four PP-MS patients showed the most prolonged time parameters (Figures 1A,C). Worth noting that the significant differences in TG parameters between MS and HS (Table 5) were observed in the presence of high correlations between time parameters, both in MS patients and in HS (Figure 2).

## Discussion

Prompted by the potential contribution of FXII in MS, in this study we provide the investigation of multiple FXII-related variables, to better define the relation between FXII and disease. This approach was coupled with global evaluation of the intrinsic pathway, with FXII activation obtained by artificial and natural molecules. Our main aims were to reveal differences between MS patients and HS, among MS clinical phenotypes, and in addition to evaluate in MS patients the variation over time of FXII-related variables.

The investigation on FXII:Ag revealed significantly increased levels in MS patients. FXII:Ag provides information about the concentration of circulating FXII protein independently from its activation and activity, presence of inhibitors, and other factors participating in the coagulation pathway. We did not observe, even as a trend, higher levels of FXII:c in RR-MS and SP-MS patients compared to HS as reported by a previous study (14). However, our cohorts were smaller (with exception of the PP-MS group) than those of the German study and, in accordance with our study design, we did not investigate FXII:c during relapse. Further comparison between data in German and Italian MS patients is hampered by absence of information about FXII protein levels (FXII antigen) in German patients. Nevertheless, the increased FXII:Ag levels detected in Italian MS patients and the increased FXII:c detected in German MS patients are both candidate to increase FXII-related immunomodulatory function. Of note, both FXII protein forms, the zymogen and the active ones, would express the immunomodulatory role independently from FXII activation in the coagulation pathway.

Repeated evaluation over 4 months of FXII:c, FXII:Ag levels, and FXII:ratio were instrumental to investigate their variation overtime in patients. We observed high correlation among time points for each FXII parameter. This feature could support a meaningful investigation of FXII contribution to disease phenotype and progression in future prospective studies.

Interestingly, FXII:c displayed a trend for variation across the time points. This could highlight changes dependent on the rehabilitative treatment, as inferred by comparison of FXII:c at T0 and T1 time points, as well as independent from treatment, as inferred by measurements prior and after 3 months of the rehabilitative training program (T0 vs. T3). Aimed at improving knowledge about the FXII role in the disease, we provided quantitative information about the FXII procoagulant activity in relation to the amount of circulating protein, by evaluating their ratio. This analysis indicated a significantly lower FXII:ratio in MS patients. This novel finding prompted us to investigate in selected groups of MS patients and HS the intrinsic pathway by TG, which provides kinetic information and potentially mechanistic interpretation of differences. Coherently with the decreased FXII:ratio, we report in MS patients a trend of lower amounts of thrombin potential, ETP, a stable and highly affordable parameter.

We introduced in this study a modified TG assay with double activation, obtained by the addition of NA, which has been recognized among true physiological activators of the contact pathway (31, 32).

Interestingly, NAs released from dead and dying cells may induce an autoimmune response by activating specific sensing receptors (33), thus representing candidate molecules of the complex crosstalk between coagulation pathway, inflammation, and immune system.

Additional trigger by NA shortened time parameters less in MS patients as compared with HS. Overall, the lower FXII:ratio and longer TG time parameters suggested that in part of MS patients (i) FXII could be less active per antigen unit and (ii) FXII response to contact activation and its support to the intrinsic coagulation pathway could be reduced. Interestingly, it has been recently reported that in TG, triggered by extrinsic activation, time parameters were shorter in MS patients (23), which does not conflict with our data because extrinsic TG does not explore FXII contribution. Noteworthy, both the intrinsic TG, first reported in our study, and the extrinsic TG (23) tightly depend on activation and activity of coagulation factors in the common pathway, essential to generate thrombin. Although indirectly, our study does not support the presence of a prothrombotic state in the MS patients under study.

In light of the increased FXII protein levels and decreased activation that we report, pharmacological inhibition of FXII, proposed as a potentially new approach to MS treatment, needs deep investigation.

The low number of patients under DMTs and the extremely heterogeneous DMTs did not permit us a productive analysis of FXII levels in relation to DMTs. Nevertheless, these study features enabled us to obtain FXII-related values reasonably independent from drugs, like interferon that is known to heavily influence gene expression in several tissues. These values could better reflect the “biological” relation between FXII and (untreated) disease. On the other hand, the investigation of DMTs effects on FXII-related variables in a properly designed study would provide a comprehensive picture of this poorly defined field.

In conclusion, our study points toward FXII-related differences between MS patients and HS, with the limitation of the small sample size. Multiple specific and global coagulation assays could help stratification of patients to better define FXII contribution to disease phenotype and progression.

## Ethics Statement

This study was approved by the Ethics Committee of Ferrara province with approval number 101-2012. Written informed consent was obtained from all participants.

## Author Contributions

NZ, MB, GM, and FB conceived the study design and wrote the manuscript; NZ, RV, and PS collected plasma samples and evaluated pre analytical variables; NZ and MB set up ELISA and aPTT; NZ performed ELISA, aPTT, and analyzed data; RM set up and performed aPTT; MB performed thrombin generation and analyzed data; SS, FM, PZ, and NB designed and supervised the rehabilitation study, recruited patients, and performed their clinical evaluation; SS and EM collected and analyzed instrumental and clinical data for patients classification. All authors critically evaluated the manuscript.

## Conflict of Interest Statement

The authors declare that the research was conducted in the absence of any commercial or financial relationships that could be construed as a potential conflict of interest.
